# Evidence‐Based Behavioral Interventions to Improve the Double‐Duty Actions Impacting the Dual Burden of Malnutrition: A Scoping Review

**DOI:** 10.1002/fsn3.71161

**Published:** 2025-11-10

**Authors:** Mary O. Hearst, Elizabeth Weinfurter, Lillian Norman, Clara A. Normile, Hussen Mekonnen, Melissa N. Laska

**Affiliations:** ^1^ School of Nursing University of Minnesota Minneapolis Minnesota USA; ^2^ Health Sciences Library University of Minnesota Minneapolis Minnesota USA; ^3^ Hubert H. Humphrey School of Public Affairs University of Minnesota Minneapolis Minnesota USA; ^4^ University of Colorado Anschutz Aurora Colorado USA; ^5^ College of Health Science Addis Ababa University (AAU) Addis Ababa Ethiopia; ^6^ Division of Epidemiology and Community Health, University of Minnesota Minneapolis Minnesota USA

## Abstract

There are three double‐duty actions that prevent both stunting and obesity, the dual burden of malnutrition, that can be implemented at health clinics worldwide. They are breastfeeding, young child nutrition, and antenatal care and women's nutrition. There is no scoping review that provides a summary of evidence‐based interventions that increase the double‐duty actions. Inclusion criteria included articles from 2010 to 2024, included interventions to improve breastfeeding, young child nutrition, and antenatal care, did not restrict the intervention to supplementation alone, and were set in sub‐Saharan Africa or Asia. Using keyword search across platforms, two reviewers screened abstracts, full papers, conducted and validated data extraction using Covidence. Thirty‐six studies were included in the final analysis. Interventions included facility‐based and community‐based education and mentoring. The interventions successfully recruited pregnant women and women with young children in community settings. The most promising practices for successfully increasing breastfeeding best practices, young child nutrition, and antenatal care included training of health extension workers and peer mentors to support education and social support through community‐based women's groups and home visiting. Implementation science methods should be explored for context‐specific adaptation and implementation.

## Graphical abstract

No scoping review summarizes evidence‐based behavioral interventions that improve double duty actions to prevent stunting and obesity. Inclusion criteria included articles from 2010 to 2024, behavioral interventions to improve breastfeeding, young child nutrition, and antenatal care, and were set in sub‐Saharan Africa or Asia. The most promising practices for increasing breastfeeding, young child nutrition, and antenatal care involved training of health extension workers and peer mentors to provide education and social support in the community along with women's groups.

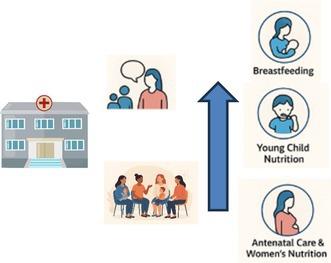

## 1 Introduction

The co‐occurrence of under and overnutrition among individuals at the household level and at the population level—commonly known as “double burden of malnutrition” (DBM)—is a growing challenge globally, particularly for countries experiencing nutritional transition such as those in sub‐Saharan Africa and southeast Asia (Central Statistical Agency 2017; World Obesity 2023; Kassie et al. 2020; Tadesse et al. 2017; Mengesha et al. 2021; Kinyoki et al. 2020). Stunting, or impaired linear growth for age, is a symptom of chronic malnutrition and results in a risk of lifelong negative consequences for brain development (Fuglestad et al. 2013; Johnson 2000; Georgieff, n.d.; Kroupina et al. 2015; Heckman 2006) and poor economic outcomes (Heckman 2006; Currie and Almond 2011). Countries in a nutrition transition are continuing to experience substantial rates of stunting attributed to nutritional gaps of pregnant women and children under the age of 3 years and an influx of processed, calorie‐dense foods entering the market (Tadesse et al. 2017; Mengesha et al. 2021; Currie and Almond 2011; UNICEF W The World Bank 2021). Alternatively, overnutrition, or obesity, is associated with multiple chronic diseases including Type II diabetes, hypertension, heart disease, certain cancers, and other conditions (Kinyoki et al. 2020; Ambachew et al. 2020; Biru et al. 2021) and is rapidly increasing in sub‐Saharan Africa and southeast Asia across age groups (Ng et al. 2014; Abarca‐Gómez et al. 2017). National policy must take up the challenge of balancing activities that further reduce the incidence of stunting while simultaneously addressing rising rates of obseity (Pradeilles et al. 2019).

Three evidence‐based interventions (World Health Organization 2017a) to improve under and overnutrition—called “double‐duty actions”—include locally adapted and healthy system promotion of: (1) breastfeeding (Ma et al. 2020; World Health Organization, United Nations Children's Fund (UNICEF) 2021), (2) healthy feeding practices for young children (Tadesse et al. 2017; World Health Organization 2016; Bliznashka et al. 2021; Jaacks et al. 2017), and (3) maternal nutrition and antenatal care (ANC) (World Health Organization 2016) and can be implemented through the healthcare system. Specifically, breastfeeding (early initiation, prelacteal feeding, exclusive BF for 6 months) is associated with reduced child stunting, prevention of future obesity and regulates maternal weight loss following pregnancy to reduce maternal obesity (Ma et al. 2020; Rollins et al. 2016; Nafista et al. 2023). Complementary food introduction that includes achieving dietary diversity, adequate meal frequency, and minimally adequate diet is associated with reduced stunting in children from 6 to 36 months of age (Bhutta et al. 2013; Mennella and Trabulsi 2012). ANC and maternal nutrition (ANC visits, skilled providers, and iron supplementation) are key for healthy weight gain during pregnancy and healthy infants (World Health Organization 2017b). To support ongoing efforts to promote healthy pregnancy weight, birth outcomes, and child growth (Jaacks et al. 2017), attention is needed to mitigate the DBM and associated factors, particularly as new, inexpensive food products are introduced (Dagne et al. 2019; Popkin et al. 2020) and more women enter the workforce.

While the evidence is strong that the double‐duty actions prevent the DBM, there is no review of the literature that provides best practices for behavioral interventions to increase the prevalence of best practices in breastfeeding, young child nutrition, and ANC and maternal nutrition. Low‐ and middle‐income countries in Africa and Asia have the highest prevalence of DBM. While supplementation using specifically formulated foods for complementary feeding of children over the age of 6 months has shown some nominal improvements in dietary diversity (Technical University of Kenya et al. 2020), including micronutrient deficiency (Somassè et al. 2018), and adequate diet (The Chilenje Infant Growth, Nutrition and Infection (CIGNIS) Study Team 2010), stronger evidence is required for the impact on stunting (The Chilenje Infant Growth, Nutrition and Infection (CIGNIS) Study Team 2010; Dewi and Mahmudiono 2021) and fortification requires resources including linkages with laboratories (Technical University of Kenya et al. 2020), can be cost prohibitive, and challenging to implement at a population level without significant investment up front. Additionally, food fortification is relevant for undernutrition but does not address the issues related to overnutrition. Thus, they fall outside the scope of this paper, which focuses on dual action drivers. Therefore, the purpose of this scoping review is to analyze peer‐reviewed published works that describe behaviorally based interventions in sub‐Saharan Africa and SE Asia focused on increasing prevention practices in the double‐duty actions to prevent DBM.

## 2 Methods

We used the methodological framework for scoping studies described by Arksey and O'Malley (2005) to map relevant literature in the field. A scoping review is an appropriate method to map the volume, nature, and characteristics of a diverse body of existing literature (Arksey and O'Malley 2005). Arksey and O'Malley note that the scoping review process is iterative in nature, and it often uses broad terms and few limitations at the beginning of the process to gain a fuller understanding of the shape of the published literature. As the shape of the literature emerges, decisions are made about search terms and limitations to best align with the objectives of the study. Our protocol was drafted using the Joanna Briggs guidance for scoping reviews (Peters et al. 2015) and refined by the research team based on the Preferred Reporting Items for Systematic reviews and Meta‐Analyses extension for scoping reviews checklist (Tricco et al. 2018). The final protocol is registered with the Open Science Framework (https://doi.org/10.17605/OSF.IO/VQ6WG).

### 2.1 Eligibility Criteria

To be included in the review, studies needed to:

- Report on an intervention related to a double‐duty action (breastfeeding, young child nutrition, or ANC and maternal nutrition),
- Be based in sub‐Saharan Africa or Southeast Asia,
- Be published in a peer‐reviewed journal between the period of 2010–2024,
- Written in English,
- Involve human participants,
- Describe an intervention aimed at changing knowledge, attitudes and/or practices related to the double‐duty actions,
- Use quantitative or mixed method study design.

Papers were excluded if:

- Study designs were qualitative, descriptive, study protocols, reviews, small or focused subsamples that may not be generalizable,
- They focused on food formularies and supplementations to increase nutrition rather than preparation and use of locally available food to increase nutritional content.

### 2.2 Information Sources and Search

**FIGURE 1:**
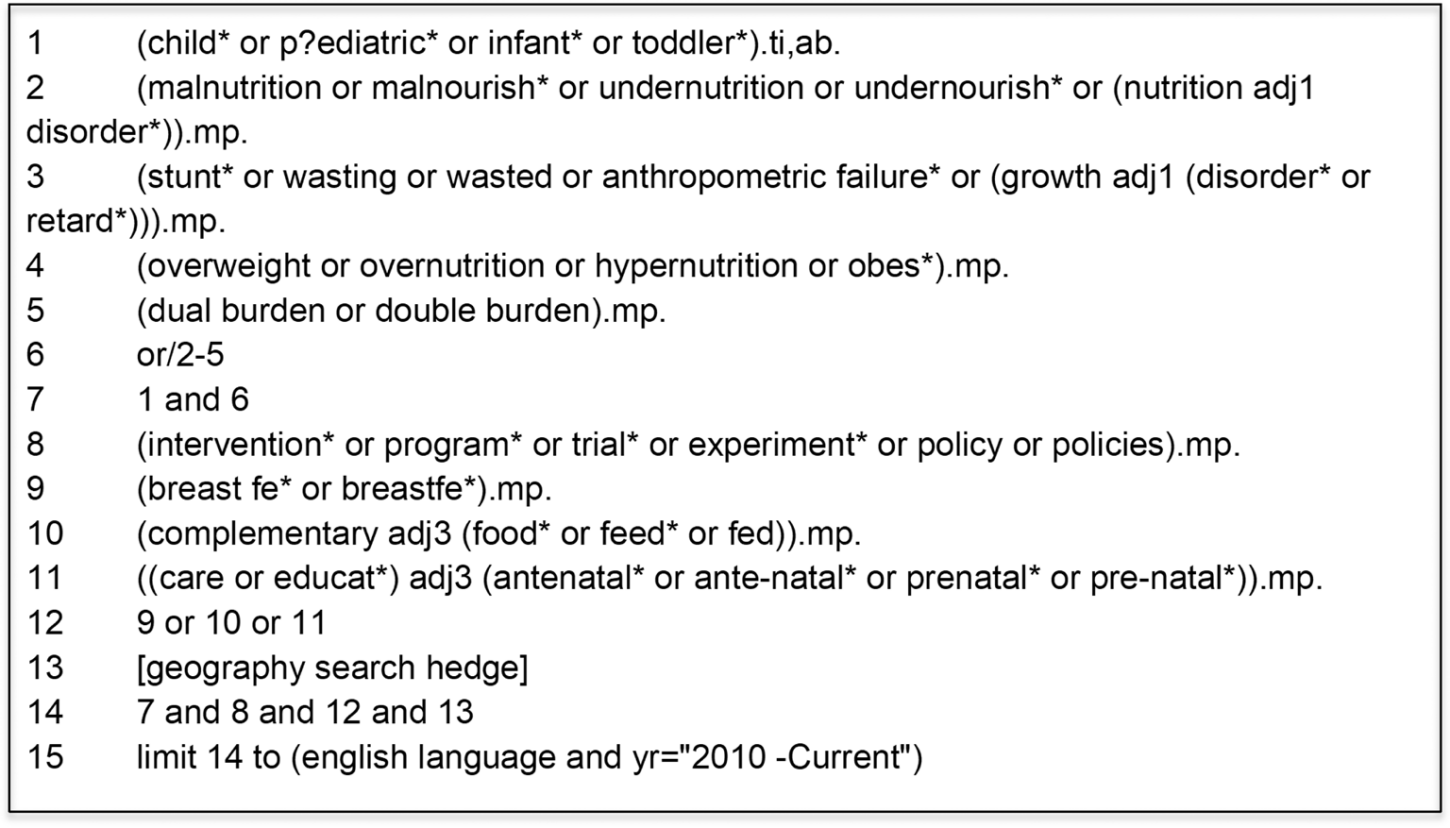
Search strategy.

Iterative pilot searches, validation against known articles, and team discussions resulted in a final search approach that combined the concepts of dual burden, the double‐duty interventions (BF, YCN, and ANC), and the geographic regions of interest (Figure 1). The librarian team member (EW) developed and validated the final search strategy, with input from team members, and the literature search was conducted on October 23, 2024 in the following databases: MEDLINE, Agricola, and CAB Abstracts via Ovid, and Scopus. Databases were selected to provide broad coverage of health sciences, agricultural and food systems literature and to capture relevant studies from these disparate disciplines. Complete search strategies are available in Appendix 1. Results were limited to English language articles published from 2010 to 2024. The 2010 start date was used because there were multiple national demographic and health surveys completed with publicly available surveillance data related to this topic and included the densest number of articles available. The strategy was optimized for each database, and a search hedge, similar to a search filter but related to subject materials, based on existing strategies (https://guides.library.ualberta.ca/health‐sciences‐search‐filters/geography) for retrieving literature in the desired geographic areas was constructed. We also conducted a hand search of reference lists of included studies to identify any articles not captured through the database search. De‐duplication and screening of references was conducted using the Covidence systematic review software platform (Covidence 2024).

### 2.3 Selection of Sources and Evidence

Two team members independently evaluated all identified papers using the screening criteria based on the initial review of titles and abstracts. Following this step, two team members reviewed full‐text versions to further screen articles based on inclusion and exclusion criteria. Disagreements were resolved in discussion. Following this screening and discussion of fit with the purpose of the scoping review, consensus was achieved on a final set of articles for inclusion in the charting step.

### 2.4 Data Charting

A data‐charting form, or a template for data extraction, in Covidence (2024) was prepared by the lead author and pilot tested by two reviewers to confirm which variables to extract. The two reviewers independently charted the data, reviewed the results, and continuously updated the data‐charting form in an iterative process. Validation of differences in charting included review of the original article and updating the charting. Further refinement occurred upon extraction to table format.

## 3 Results

**FIGURE 2:**
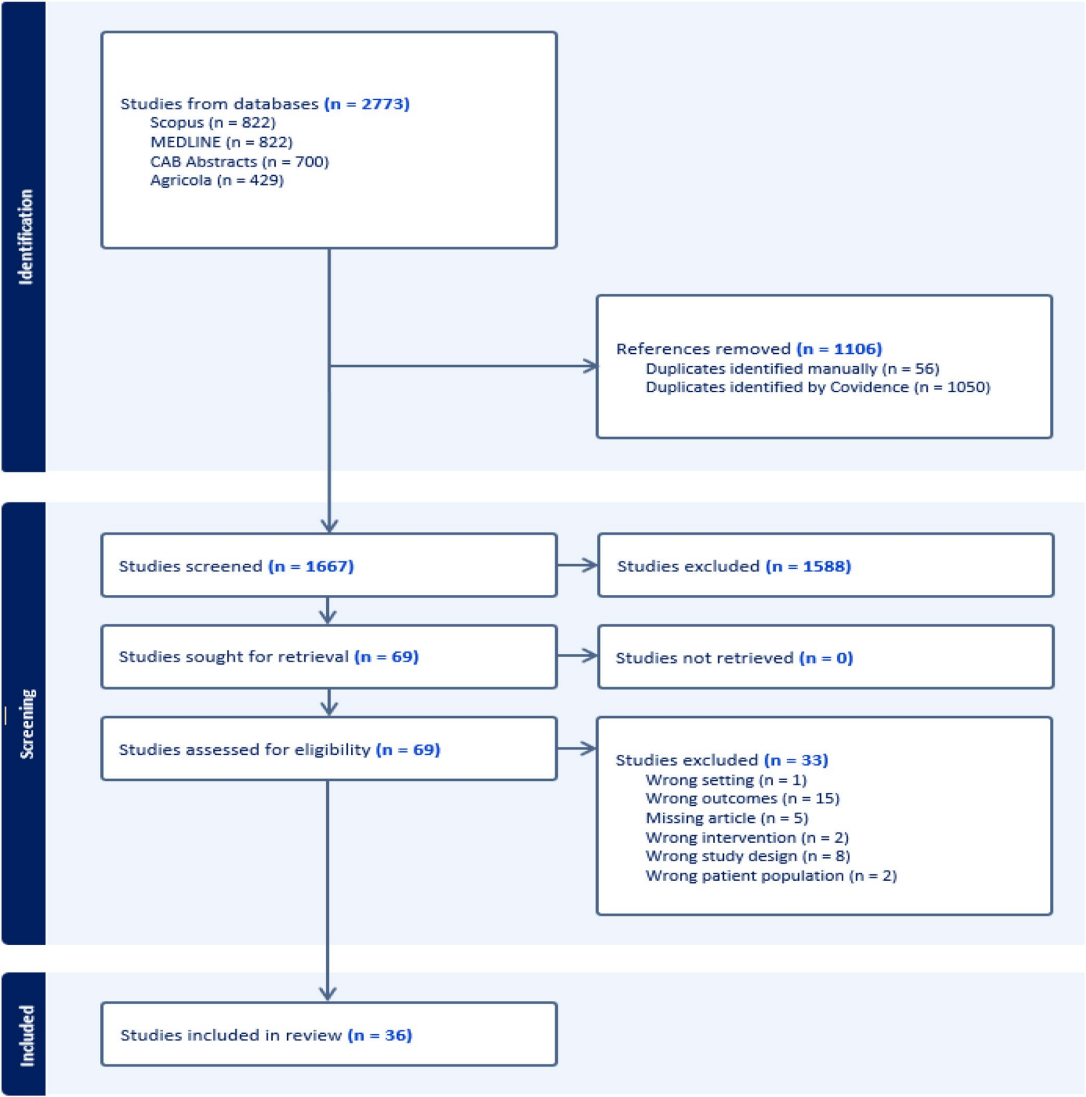
PRISMA flow diagram of search results.

A total of 36 papers met the inclusion criteria and are summarized in Tables 1 and 2. The PRISMA flow diagram of search results is included in Figure 2.

**TABLE 1:** Table of included nonrandomized studies with intervention focus on changing breastfeeding, young child nutrition, and antenatal care and maternal nutrition behaviors.

Author, date	Country	Topic	Population	Intervention	Key findings	(+/−/0)
Dearden, 2023	Tanzania	BF, ANC	14,999 mothers; 6726 fathers with children under 2 years of age	The program trained more than 6000 community health workers, facility workers, staff, and volunteers to encourage cultural and behavioral shifts in maternal child nutrition practices. Mass media included ratio and television spots, interpersonal communication was completed at home visits and organized mobile outreach. Group‐based approaches included positive deviance/hearth groups and support groups. Baseline, midline, and endline data collection.	Women's knowledge of when to start complementary foods increased. Women reported attending more antenatal care visits, more early initiation of breastfeeding from baseline to midline. Men increased the number of times they fed the youngest child. Knowledge of early initiation and exclusive breastfeeding for the first 6 months did not change.	Mixed
Desai, 2015	Zimbabwe	BF, YCN	19 mothers who had infants 5–9 months of age	Village health workers trained for 2 weeks and delivered five modules to mothers over 5 weeks. Each module included tools and/or activities to illustrate key concepts.	Maternal knowledge increased after each module. Nutritional adequacy improved.	Positive
Fahmida, 2015	Indonesia	YCN	494 mothers with a child aged 9–16 months	The intervention was a 4‐part factorial design of complementary feeding recommendations (CFR) and psychosocial stimulation. Comparison was made only between CFR and non‐CFR groups. Monthly sessions were delivered by a study team member and assisted by the subvillage coordinator and voluntary health workers. Sessions included demonstration and practice, strategies to increase diversity with local foods, and games.	Maternal knowledge improved in the CFR group compared to non‐CFR. MDD improved significantly in the CFR group.	Positive
Fiorella, 2019	Kenya	YCN	223 primary caregivers and 227 children 1–3 years of age	Training of community health workers to involve mothers, fathers, and grandparents in nutrition education and strategies to provide support in the community. The intervention was six sessions over 12 weeks delivered by community health workers and included knowledge and activating social support using hands‐on activities, dramatizations, and demonstrations.	Knowledge among community members and community health workers was maintained for 2 years post intervention. Immediately postintervention, children in the intervention group had higher MMF, then returned to baseline at 6 months.	Null
Kartasury, 2019	Indonesia	YCN	80 pairs of mothers and children under 2 years	Ten cadres (community health workers) were trained in complementary feeding practices. Each cadre met with 2 mothers for 2 months including education, recipes, a handbook, and demonstrations.	Mother's attitude toward feeding nutritious food and feeding practices were better in the intervention group.	Positive
Kimani‐Murage, 2016	Kenya	BF	Pregnant women and babies from 0–6 months. Pre‐intervention (2007–2011; *n* = 5824), Intervention (2012–2015; *n* = 1110) and Comparison (2012–2014; *n* = 487)	The intervention was personalized home‐based visits by trained community health workers. Visits began during pregnancy (monthly), then weekly after 34 weeks until birth, then monthly. Topics including maternal nutrition, early initiation of breastfeeding, duration of breastfeeding.	EBF was higher in the intervention study, but not in the comparison study.	Positive
Kung'u, 2018	Ethiopia; other (place into notes)	BF, ANC	8765 women and children 0–11 months across three countries	All three country's interventions included behavior change interventions, training of community health workers, and organizational systems and supports.	No significant differences across countries in ANC indicators except the first ANC visit in the first trimester per country. Iron and folic acid consumption was higher in the intervention group in each country but only more than 90 days in Kenya. Women in the intervention condition were more likely to receive counseling for breastfeeding practices but mixed for ANC. Early initiation improved in Ethiopia, not Senegal. EBF intervention effect in Kenya. Facility based delivery improved for all countries.	Mixed
leRoux, 2020	South Africa	ANC, EBF	1310 pregnant women	Women whose children are thriving were recruited and trained to work as a community health workers in the area they live. They do house‐to‐house visits, identify maternal and child health issues, and enroll families into regular home visits. Counseling includes ANC, breastfeeding, and child health.	Antenatal visits were higher, and exclusive breastfeeding rates at 6 months were higher (especially among HIV negative women) and mothers were less likely take their children to a traditional healer.	Positive
Maidelwita, 2022	Indonesia	YCN	72 mothers with babies aged 6–12 months	Nutritional counseling at the health center with a demonstration of complementary feeding compared to only nutritional counseling, focused on children 6–12 months of age	The study found that there are significant differences between the nutritional counseling plus demonstration with conventional nutritional counseling.	Positive
Marah Has, 2023	Indonesia	YCN	60 mothers and their youngest child 6–11 months	An 11‐week family empowerment intervention delivered in the community delivered once a week for 9 weeks in groups, comprised of 4–6 people living in one neighborhood.	Significant increases in MDD, MMF, MAD at the post test in the intervention groups compared to control group (no baseline differences).	Positive
Nafista, 2023	Indonesia	YCN	70 mothers	The intervention participants received young child feeding using a flipchart developed by the WHO, two sessions about complementary feeding for 30–40 min each, and the nutrition education was delivered by the researcher and a certified dietitian from the local community healthcare center within the village for 5–7 mothers. The control group received usual care from the Indonesian maternal and child handbook and the same two education sessions.	Mothers' knowledge and attitudes were higher in the intervention compared to control group.	Positive
Namukose, 2024	Uganda	ANC	784 Pregnant mothers and infants followed for 12 months postdelivery	The intervention group received a comprehensive nutrition assessment counseling and support compared to usual care. Health workers completed a 5‐day training program. They were provided with anthropometric equipment, policy guidelines, job aides, information, education, and communication materials. Services ranged from linking mothers and their infants to community support structures to ensure ongoing nutrition care and support, monitoring, nutrition interventions, education, supplementation, and follow‐up. The usual care received growth monitoring and promotion, supplementation, and training and educational supplies for staff.	Maternal dietary diversity and iron/folic acid supplementation significantly improved in the intervention compared to usual care group.	Positive
Olusa, 2023	Nigeria	YCN, BF	240 pregnant women in third trimester	The intervention comprised of 3 months training of recruited pregnant women followed by 12 months of peer support groups (15 members) intervention of the same set of mothers with their infants. The participating pregnant mothers received the conventional antenatal care and education. The experimental LGA had nine peer support groups and these support groups were motivated to meet once every month. Home visits were periodically conducted by the support group leaders to overcome barriers. The control group received usual antenatal care education but they were not aided by the formation of IYCF support groups within their communities.	Peer support group counseling increased breastfeeding initiation, lowered prelacteal feeding, increased exclusive breastfeeding for 6 months, timely introduction of complementary foods, and MDD.	Positive
Rana, 2018	Vietnam	BF, YC	376 households with children under 2 years	Support groups were formed and facilitated by trained staff including a monthly breastfeeding group, monthly complementary feeding, and community support group for fathers, grandparents, and village leaders compared to households in communities where they did not occur.	Knowledge of breastfeeding and complementary feeding was higher where the support groups existed. MDD was significantly higher with the intervention but not breastfeeding practices or other YCN practices.	Mixed
Saaka, 2021	Ghana	YCN	712 mothers with children aged 6–36 months	The intervention consisted of a series of recorded health and nutrition drama that were broadcast once a week for a 12‐month duration in local dialects on five radio stations. The radio material used was recorded drama series whose content included nutrition for pregnant women and lactating mothers, appropriate CF practices, planning diversified diets for the household, active feeding, the importance of consumption of fruits and vegetables for health and different ways men can help their wives to improve nutrition in the family.	Knowledge, attitudes, and practices were higher than comparison group. MDD significantly increased compared with the comparison group.	Mixed
Tessema, 2023	Ethiopia	YCN	2160 households with children under 2 at baseline; 2356 households at endline	Multisectoral government led program integrating health and agriculture service delivery that included individual, social, organizational, community, and policy spheres. Standard care of nutrition services by health extension workers. Enhanced intervention included counseling for couples, women and men's group dialogues in the community, demonstration gardens, broadcast messages, and poultry or seeds for lowest socioeconomic decile.	Women's knowledge of dietary diversity needs increased. Little improvement in agricultural production per household. MAD, MMR and MDD increased.	Positive

Abbreviations: ANC, antenatal care and maternal nutrition; BF, breastfeeding; MAD, minimal acceptable diet; MDD, minimum dietary diversity; MMF, minimum meal frequency; YCN, young child nutrition.

**TABLE 2:** Table of included randomized studies with intervention focus on changing breastfeeding, young child nutrition, and antenatal care and maternal nutrition behaviors.

Author, date	Country	Topic	Focus population & #	Intervention	Key findings	Outcome (+/−/0)
Beatty, 2024	Indonesia	BF; YCN; ANC	9120 Mothers; children 0–35 months	The project provided training on IYCF and child growth monitoring for health providers (village health post volunteers, village midwives, nutritionists and midwives at subdistrict health clinics, and district and provincial health officials) in communities that received a grant. Training lasted between 3 and 8 days and covered a variety of technical topics related to breastfeeding, complementary feeding, growth monitoring, and women's nutrition. Service providers applied what they learned in village level nutrition counseling for pregnant women and for caregivers of children under 5 years old. Growth monitoring equipment was provided and folic acid and iron supplementation.	Intervention mothers consumed more iron and folic acid during pregnancy, were more likely to exclusively breastfeed 0–5 month old infants, and more 6–23‐month‐old children achieved the recommended MMF. ANC visits, MDD did not improve.	Mixed
Effendy, 2020	Indonesia	YCN	242 Caregivers; children 6–17 months	The intervention groups received standard health and nutrition care plus nutrition education intervention, whereas the participants in control groups only received standard health and nutrition care from the Posyandu. Standard care for children under 2 included child growth monitoring, nutrition counseling, basic immunizations, Vitamin A supplementation and fortified biscuit for underweight children. The nutrition education intervention provided 4 nutrition classes and once monthly home visits for 3–4 months by trained cadres.	Children in the intervention group had higher MDD scores in the intervention compared with control group.	Mixed
Gebremariam, 2023	Ethiopia	BF	122 couples, women pregnant in third trimester	There were three arms including mothers and fathers receiving BF education through text messages in addition to standard care; mothers only received messages plus standard care; and the couples only received standard care. Text messages were aligned with BF recommendations, 16 weekly messages included milestones and issues. Messages tailored to mothers and fathers.	Exclusive BF was higher at months 2 and 3 for the mother–father intervention compared to control and in the Month 3 for the mother only intervention. Mean differences in breastfeeding knowledge scores for mothers in the MFI, and MI intervention groups, mothers attitudes in the MFI group compared to mothers in the CG, support mothers in the MFI group indicated that they received better support in terms of breastfeeding “savvy” than the other groups.	Positive
Han, 2019	Ethiopia	YCN	510 Women with at least 1 child 4–20 months, men who lived with women more than 9 of the last 12 months.	All treatment arms included a maternal behavioral change communication (BCC) program lasted for 16 weeks. Seven to 14 participants from the same garee (village) formed 1 BCC group and met with a trained facilitator at the nearest health posts once a week for an hour. Messages included diversity, quantity, preparation, and storage of complementary food. In some treatment arms, fathers participated in a paternal BCC program that lasted for 12 weeks of 7–14 members and included messages about diet diversity, consequences of malnutrition during the first 2 years, fathers role in childcare, shared division of household labor, and gender equal intrahousehold decision. Finally, two treatment arms included food vouchers worth 200 ETB approximately US $10 that were transferred monthly to each household for 4 months.	Maternal and paternal knowledge increased. Maternal BCC increased MDD compared to the control group. When maternal BCC was combined with either paternal BCC or the food voucher, the increase in MDD was stronger. No change in MMF and MAD.	Mixed
Hewett, 2020	Zambia	YCN	2660 Adolescent girls 10–19 years	Weekly girls group meetings conducted over 2 years in which girls met with a trained mentor to discuss a curriculum‐guided topic, discuss personal matters, to socialize. Within the weekly girls' groups meetings, three core curricula were used by the mentors to guide the individual sessions: (1) health and life skills, (2) financial education and (3) a nutritional curriculum. The nutritional curriculum was tailored to provide age‐appropriate information through six weekly sessions on nutrition. Sessions were conducted over consecutive weeks and were repeated in program years one and two.	No statistically significant differences nutritional study arms were observed in identifying health foods or proper infant feeding practices.	Null
Ijumba, 2015	South Africa	BF	4137 Pregnant women	Thirty CHW from the intervention clusters were trained for 10 days on home entry, motivational interviewing techniques, feeding practices, and antenatal care. The intervention was delivered by CHW living in the clusters though a structured home visiting schedule. Each visit was designated to cover specific topics related to the outcomes of the study. Visits in the intervention arm included two home visits during pregnancy, one in the f irst 48 h after delivery, then at 3–4, 10–14 days, 3–4 weeks and a final visit at 8–9 weeks. All neonates with low birth weight received two extra visits during the first week. Control cluster package CHW living in control clusters provided essential information and support to pregnant women on how to obtain state social welfare grants. Visits in the control arm included one home visit during the antenatal period and two postnatal visits at 4–6 weeks and 10–12 weeks.	There was no difference in the timing of initiation of breastfeeding between study arms. Exclusive BF was doubled in the intervention arm at 12 weeks, exclusive formula‐feeding increased, and predominant breast‐feeding also increased, and mixed formula‐feeding as well as mixed breast‐feeding were reduced.	Mixed
Kang, 2017	Ethiopia	YCN	2064 mothers of children 6–12 months	Twenty‐three volunteers were recruited and trained from local communities along with nine supervisors. The intervention was the addition of a community participatory nutrition program to usual care. Mothers participated in a 2‐week, 12 daily group session (7–12 mothers each group) that included promoted IYCF practices such as optimal breast‐feeding, introducing varied and nutritious food groups in complementary foods to improve dietary diversity, and hygiene behaviors including individual and environmental hygiene practices, and demonstrations. After the two‐week session, 1–2 home visits were made by the volunteer or supervisor.	The MMF was higher in the intervention compared to control condition. No difference with current breastfeeding, MDD, or hand washing. Composite feeding scores (DD + MF + BF) and (DD + MF) were significantly higher in the intervention v control conditions.	Mixed
Lewycka, 2013	Malawi	BF; ANC	43,719 women aged 10–49 years	Seventy‐two volunteer peer counselors were trained and made home visits five times. Women were in four arms: women's group and volunteer peer counseling, women's group only (20 sessions), peer counseling only (5 visits), no intervention. Women's groups were organized and discussed maternal and child health problems and strategies. Peer counseling included five home visits during and after pregnancy and included support and counseling.	Women's groups mobilizing communities improved EBF. Peer counseling is less effective without women's groups present.	Positive
Mardani, 2024	Indonesia	YCN	160 mothers with children under 2 years	Women received a 4‐week nutritional education program or usual care. The educational program included two nutritional educational sessions in the first and second weeks and two supportive phone calls for counseling in the third and fourth weeks. All participants received a booklet that included management of undernutrition and information on complementary feeding practices.	Mothers in the intervention group improved knowledge, self‐efficacy, and complementary feeding practices compared to the control group.	Positive
Mbuya, 2019	Zimbabwe	BF	2442 pregnant women; 2728 women with children under 3 months	The breastfeeding intervention included 4 modules delivered at 4 time points (7 months gestation, 3 days, 1 month, 3 months) by community health workers; each module was about 1 h in duration, and available family members were invited to participate.	Early initiation prevalence was higher in intervention participants. EBF prevalence did not differ at 1 month and declined in both groups over time; although remained higher in intervention participants	Positive
Muluye, 2020	Ethiopia	YCN	200 mothers of children 6–24 months in daycare institutions	Trained nutritionists provided face‐to‐face biweekly education for 4 months including active lecturer, posters, note pad, brochures, and practical demonstration sessions. Key messages included complementary food recipes and preparation, appropriate amount and frequency of feeding, personal hygiene and sanitation, and dietary diversification.	Maternal knowledge and complementary feeding practices were higher in the intervention group at endline compared to the control condition.	Positive
Nikiema, 2017	Burkina Faso	BF; YCN	2253 months from pregnancy to 18 months after birth	This facility‐based intervention provided patient‐centered communication and nutrition counseling training. The control group received no additional training. The training was focused on communication between care providers and women at any contact for prenatal visits and children's services; and to enhance the nutrition component of the existing maternal and child national program.	Intervention participants had more counseling and higher EBF, MMF, MDD compared to the control arm.	Positive
Ochola, 2012	Kenya	BF	360 Mothers with children 0–6 months	Three females were recruited and trained as breast‐feeding counselors including benefits, preparation for, and initiation and management of breastfeeding. The control group received the usual nutrition education in a group session. The mothers in the facility‐arm received one 30–40‐min session of one‐on‐one counseling at the Health Centre with no further intervention. The home‐based intervention arm, were visited in their homes where they received a total of seven counseling sessions beginning prenatally, then the second during the first week after delivery; and the third to seventh monthly up to 5 months postpartum.	At 1 month, EBF was higher in facility and home‐based intervention groups compared to control. Although EBF dropped overall at 3 months, the home‐based intervention mothers remained significantly higher than the control group and higher than the facility.	Positive
Osaki, 2019	Indonesia	BF; YCN; ANC	454 pregnant women	Government Maternal Child Health Booklets were provided to facilities and distributed to pregnant women, health facility staff and volunteers received orientation and job aids to support women at the facility or in the community. Monthly staff meeting monitored the use of the materials.	The intervention arem attending more ANC appointments, and had professional childbirth care. No difference in EBF, item specific MDD increased compared to control.	Mixed
Qureshi, 2011	Nigeria	BF	179 breastfeeding mothers	Ten female volunteers were trained at a 4‐day workshop comprising of lectures, role plays and demonstrations using posters and flip charts. Each session lasted two and a half hour. Training content covered counseling skills, the basics of nutrition, exclusive breastfeeding and the survey instrument. Counseling was provided to women who were breastfeeding.	After counseling, the proportion of mothers with intention to EBF increased significantly. Follow‐up suggests potential impact on EBF.	Positive
Rawat, 2017	Vietnam	YCN	1500 mother–child dyads under 59.9 months	The intervention included interpersonal counseling (IPC), mass media (MM), and community mobilizing (CM). The aim was to assess the difference between all three interventions compared to less‐intensive combinations on complementary feeding. Less intensive included lack of structure for CM, reduced MM, IPC communication as usual care.	In the intent to treat analyses, there were no significant differences between groups in changes in complementary feeding practices over time.	None
Siswati, 2022	Indonesia	YCN	60 mothers with children 0–59 months	Community volunteers were trained about communicating behavior change, growth, and development of toddlers, stimulating growth and development, reading growth charts, and providing appropriate food. The control group were only reminded to read MCH books. Trained volunteers visited mothers once a week for 4 weeks. Intervention mothers received a complementary feeding intervention consisting of carbohydrate‐source foods, animal diet, protein, cereals, vegetables, and fruits. In the control group, the health visit was only to remind mothers to read MCH books and to supply complementary feeding.	The intervention arm had greater gain in knowledge scores than the control group. Practices were significantly higher in intervention versus control however it is not known what the practices were.	Mixed
Tariku, 2015	Ethiopia	YCN	166 mothers with children 6–18 months	The intervention included typical training by health extension workers but in context of malnutrition susceptibility, severity, benefits of appropriate complementary feeding practices, and self‐efficacy to prepare complementary feeding plus barriers. Control participants received usual care by the health extension workers. Education was provided every 2 weeks for both groups and the intervention mothers met as a group two times during the intervention period.	No intervention effect concerning continued breastfeeding duration and frequency. No effect on dietary diversity but intervention effect on meal frequency.	None
Tylleskar, 2011	South Africa; Uganda, Burkina Faso	BF	2579 mothers; children 0–6 months	Peer counselors living near the intervention clusters were recruited and trained for 1 week. The peer counselors offered home‐based‐breastfeeding peer support. Mothers received at least 5 visits staring in the last trimester. Mothers and infants in control clusters in Burkina Faso and Uganda were given standard health care only, and those control clusters in South Africa were visited by peer counselors, with the same schedule as in the intervention clusters, but who assisted families in obtaining birth certificates and social welfare grants.	In all three countries, prevalence of EBF at 12 weeks of age in the intervention cluster was about twice that in the control cluster. The prevalence of EBF was lower at 24 weeks of age than at 12 weeks in the intervention and control clusters in Burkino Faso, Uganda, and South Africa, whereas the differences (prevalence ratios) were higher.	Positive
Waswa, 2015	Kenya	YCN	207 households with children aged 6–17 months	The intervention focused on 10–15 caregivers with children aged 6–17 months in the village. The nutrition education intervention consisted of four sessions consisting of group training and cooking demonstrations. The key messages and pictures on the importance of breast‐feeding, age‐appropriate complementary feeding practices, hygiene and feeding young children a variety of foods were compiled into folders, brochures and posters. In April 2013, individual home‐based follow‐up visits were made on a randomly selected sub‐sample of caregivers in the intervention villages who had participated in the first two sessions.	A significantly higher proportion of children in the intervention group compared with those in the control group achieved MDD. The proportion of breast‐fed children who achieved MMF and MAD was significantly higher in the intervention group compared with the control group at endline. There was an intervention effect on nutrition knowledge. Knowledge scores increased based on the number of sessions (4 possible) they attended.	Positive

### 3.1 Characteristics of Studies

Sixteen studies were nonrandomized (including 10 quasi‐experimental (Nafista et al. 2023; Desai et al. 2015; Kartasurya et al. 2019; Olusa et al. 2023; Maidelwita et al. 2022; Fahmida et al. 2015; Le Roux et al. 2020; Saaka et al. 2021; Marah Has and Arief 2023; Namukose et al. 2024)), 5 pre–post evaluation designs with no control group (Rana et al. 2018; Fiorella et al. 2019; Dearden et al. 2023; Kung'u et al. 2018; Tessema et al. 2023), and 1 used a multimodal evaluation design including a comparison group and cross‐sectional data (Fiorella et al. 2019) and 21 studies were randomized trials (Effendy et al. 2020; Beatty et al. 2023; Tylleskar et al. 2011; Ochola et al. 2013; Lewycka et al. 2013; Tariku et al. 2015; Waswa et al. 2015; Kang et al. 2017; Han et al. 2019; Ijumba et al. 2015; Mbuya et al. 2019; Qureshi et al. 2011; Rawat et al. 2017; Nikiema et al. 2017; Osaki et al. 2019; Siswati et al. 2022; Muluye et al. 2020; Mardani et al. 2024; Gebremariam et al. 2023; Hewett et al. 2020). Nonrandomized interventions took place in Sub‐Saharan Africa (Ghana (Saaka et al. 2021), Nigeria (Olusa et al. 2023), South Africa (Le Roux et al. 2020), Tanzania (Dearden et al. 2023), Ethiopia (Tessema et al. 2023), Uganda (Namukose et al. 2024), and Zimbabwe (Desai et al. 2015)) (each country *n* = 1), Kenya (*n* = 2) (Fiorella et al. 2019; Kimani‐Murage et al. 2016) and Asia (Indonesia (Nafista et al. 2023; Kartasurya et al. 2019; Maidelwita et al. 2022; Fahmida et al. 2015; Marah Has and Arief 2023) *n* = 5, Vietnam (Rana et al. 2018) *n* = 1). One additional study included Ethiopia, Senegal, and Kenya (Kung'u et al. 2018). The randomized studies were largely in Sub‐Saharan Africa (Ethiopia (Tariku et al. 2015; Kang et al. 2017; Han et al. 2019; Muluye et al. 2020; Gebremariam et al. 2023), *n* = 6; Kenya (Ochola et al. 2013; Waswa et al. 2015), *n* = 2); one in each of Burkina Faso (Nikiema et al. 2017), Malawi (Lewycka et al. 2013), Nigeria (Qureshi et al. 2011), Zambia (Hewett et al. 2020), Zimbabwe (Mbuya et al. 2019), and South Africa and Indonesia (*n* = 5) (Effendy et al. 2020; Beatty et al. 2023; Osaki et al. 2019; Siswati et al. 2022; Mardani et al. 2024) and Vietnam (Rawat et al. 2017; Ijumba et al. 2015) (*n* = 1). One additional study included South Africa and Uganda (Tylleskar et al. 2011).

Based on our review inclusion criteria, the intervention foci were breastfeeding practices, young child nutrition, and ANC and maternal nutrition. Of the 36 studies, 10 randomized control trials (Beatty et al. 2023; Tylleskar et al. 2011; Lewycka et al. 2013; Ijumba et al. 2015; Mbuya et al. 2019; Nikiema et al. 2017; Osaki et al. 2019) and 6 nonrandomized studies (Olusa et al. 2023; Le Roux et al. 2020; Dearden et al. 2023; Osaki et al. 2019; Kimani‐Murage et al. 2016) included breastfeeding outcomes. Young child nutrition, including dietary diversity, minimum meal frequency, and minimal adequate diet, was included in 14 randomized controlled trials (Effendy et al. 2020; Beatty et al. 2023; Tariku et al. 2015; Waswa et al. 2015; Kang et al. 2017; Han et al. 2019; Rawat et al. 2017; Nikiema et al. 2017; Osaki et al. 2019; Siswati et al. 2022; Muluye et al. 2020; Mardani et al. 2024; Hewett et al. 2020) and 11 nonrandomized studies (Bhutta et al. 2013; Desai et al. 2015; Kartasurya et al. 2019; Olusa et al. 2023; Maidelwita et al. 2022; Fahmida et al. 2015; Saaka et al. 2021; Marah Has and Arief 2023; Namukose et al. 2024; Rana et al. 2018; Fiorella et al. 2019; Tessema et al. 2023). ANC and maternal nutrition was included in three randomized controlled studies (Beatty et al. 2023; Lewycka et al. 2013; Osaki et al. 2019) and three nonrandomized studies (Namukose et al. 2024; Dearden et al. 2023; Kung'u et al. 2018). Four randomized controlled trials (Beatty et al. 2023; Ochola et al. 2013; Nikiema et al. 2017; Osaki et al. 2019) and seven nonrandomized studies (Desai et al. 2015; Olusa et al. 2023; Le Roux et al. 2020; Rana et al. 2018; Dearden et al. 2023; Kung'u et al. 2018) included multiple intervention foci.

### 3.2 Program Feasibility

Each study included in this review demonstrated feasibility for site identification, recruiting healthcare professionals, community outreach experts, and/ or families with young children and successful training of the data collection and/or intervention staff. Across the studies, the most common intervention component included maternal education (Nafista et al. 2023; Desai et al. 2015; Kartasurya et al. 2019; Olusa et al. 2023; Maidelwita et al. 2022; Fahmida et al. 2015; Le Roux et al. 2020; Marah Has and Arief 2023; Namukose et al. 2024; Rana et al. 2018; Fiorella et al. 2019; Dearden et al. 2023; Kung'u et al. 2018; Tessema et al. 2023; Gebremariam et al. 2023; Kimani‐Murage et al. 2016) (*n* = 16). Eleven studies explicitly addressed the training of outreach health workers affiliated with healthcare facilities (Desai et al. 2015; Kartasurya et al. 2019; Fahmida et al. 2015; Rana et al. 2018; Fiorella et al. 2019; Dearden et al. 2023; Kung'u et al. 2018; Tessema et al. 2023; Gebremariam et al. 2023; Kimani‐Murage et al. 2016). Eight studies stated general community education as a component of the intervention (Saaka et al. 2021; Fiorella et al. 2019; Dearden et al. 2023; Kung'u et al. 2018; Tessema et al. 2023; Han et al. 2019; Osaki et al. 2019; Gebremariam et al. 2023). Seven studies included interventions focused on fathers (Saaka et al. 2021; Rana et al. 2018; Fiorella et al. 2019; Dearden et al. 2023; Tessema et al. 2023; Han et al. 2019; Gebremariam et al. 2023), six included training and implementation of social support (e.g., mothers' groups (Lewycka et al. 2013), grandmothers (Saaka et al. 2021; Fiorella et al. 2019; Tessema et al. 2023), peers (Rana et al. 2018; Fiorella et al. 2019)), and three of the interventions focused on the health care providers at the facilities directly (Effendy et al. 2020; Mbuya et al. 2019; Osaki et al. 2019). Four studies used mass media messages to spread health promotion information (Saaka et al. 2021; Dearden et al. 2023; Rawat et al. 2017; Kim et al. 2019), and one study used text messaging (Gebremariam et al. 2023).

The authors did make suggestions for future research and key elements that should be noted. For example, it was important to pilot the feasibility of the intervention prior to implementation (Desai et al. 2015; Beatty et al. 2023; Rawat et al. 2017; Gebremariam et al. 2023). In several cases, there were hypothesized gaps in the fidelity of the implementation of the intervention, resulting in recommendations for researchers to monitor program implementation, provide adequate supervision for community outreach staff, and provide additional training as needed (Kartasurya et al. 2019; Qureshi et al. 2011; Rawat et al. 2017; Osaki et al. 2019; Kimani‐Murage et al. 2016). It was important to be aware of and mitigate barriers experienced by community outreach workers (Le Roux et al. 2020). Other recommendations included being aware of simultaneous interventions happening in the same community unrelated to the study (Le Roux et al. 2020; Saaka et al. 2021) and having the intervention be part of an existing system of care, such as the health facilities and routine services provided (Namukose et al. 2024; Rana et al. 2018; Ochola et al. 2013; Lewycka et al. 2013; Nikiema et al. 2017). Finally, it was strongly recommended that fathers, grandmothers, and other influential family or community members be integral to the intervention (Ochola et al. 2013; Waswa et al. 2015; Han et al. 2019; Ijumba et al. 2015; Mbuya et al. 2019; Gebremariam et al. 2023). Yet, before introducing fathers into a key feeding role, an understanding of gender roles was needed to avoid fathers usurping the mother's role in the family (Han et al. 2019; Mbuya et al. 2019).

### 3.3 Study Findings for Behavioral Outcomes

The results are presented for nonrandomized studies separately from the higher‐quality and more rigorous randomized control trials. The key intervention types were (1) health facility based only; (2) educational campaign; (3) community health worker outreach with education only; and (4) community health worker outreach with education and support groups.

#### 3.3.1 Breastfeeding

The most common outcome of interest across the studies was exclusive breastfeeding for 6 months, followed by early initiation and reducing prelacteal feeding.

#### 3.3.2 Nonrandomized Studies

Community outreach workers provided direct education and encouragement on breastfeeding practices starting in late pregnancy and following mothers until reaching 6 months postpartum (Desai et al. 2015; Le Roux et al. 2020; Rana et al. 2018; Fiorella et al. 2019; Dearden et al. 2023; Kung'u et al. 2018). The community outreach workers visited the women at their homes and provided education and support. Community‐centered interventions generally resulted in increased early initiation of breastfeeding (Kung'u et al. 2018) and exclusive breastfeeding (Kung'u et al. 2018; Kimani‐Murage et al. 2016), but results were not entirely consistent (Fiorella et al. 2019; Dearden et al. 2023). One intervention included the use of radio and television broadcasts of health promotion messages (Dearden et al. 2023). Two studies successfully included community support groups for fathers, grandparents, and village leaders or other strategies to engage broader social support for healthy nutrition (Rana et al. 2018; Fiorella et al. 2019); however, both studies only had an impact on knowledge of breastfeeding practices but not behavior.

#### 3.3.3 Randomized Trials

The most consistent and impactful intervention was home‐based counseling. Community outreach workers and/or peer counselors were trained in best practices for breastfeeding. Home visits were more successful at increasing exclusive breastfeeding (Ochola et al. 2013) in the intervention group versus the comparison group(s) (Tylleskar et al. 2011; Ochola et al. 2013; Lewycka et al. 2013; Ijumba et al. 2015). Lewycka et al. (2013) found that while peer counseling was effective in increasing exclusive breastfeeding, when combined with women's groups, the effect was comparably stronger than in the other intervention arms. One home‐based intervention in Nigeria assessed if using counseling by community health volunteers would improve maternal knowledge, intentions, and behaviors related to early initiation and exclusive breastfeeding. However, the study found significant differences in mothers' intention to exclusively breastfeed only between the intervention and control condition (Qureshi et al. 2011) A study in Burkina Faso focused on a randomized trial improving communication between the care providers and women at the health facility during any prenatal or child health visit. The results showed that the intervention arm received more counseling related to best feeding practices and women were more likely to initiate breastfeeding earlier, exclusively breastfeed, and children older than 6 months had improved dietary diversity and meal frequency (Nikiema et al. 2017). Finally, an intervention in Indonesia emphasized the use of a Maternal Child Health Booklet, and orientation and job aids for health facility staff that resulted in more knowledge among mothers but had limited intervention effects on breastfeeding (Osaki et al. 2019).

In summary, the best intervention to improve breastfeeding practices was a combination of counseling by trained community health volunteers combined with women's groups where messages were reinforced and women received social support. Second, improving care counseling at the health facilities during prenatal and well‐child visits can also be effective. Health education materials are not an effective intervention alone.

#### 3.3.4 Young Child Nutrition

The most common outcomes were increasing minimum dietary diversity, minimum meal frequency, and minimum adequate diet through general education, cooking demonstrations, and using local foods to enhance the nutritional quality of meals.

#### 3.3.5 Nonrandomized Studies

Using community outreach, maternal knowledge or attitudes of young child nutrition needs improved (Nafista et al. 2023; Desai et al. 2015; Kartasurya et al. 2019; Fahmida et al. 2015; Rana et al. 2018; Fiorella et al. 2019); however, minimum dietary diversity (Desai et al. 2015; Marah Has and Arief 2023; Rana et al. 2018), dietary nutrients (Fahmida et al. 2015) and complementary feeding practices (Olusa et al. 2023; Marah Has and Arief 2023) improved with the community outreach intervention, in some cases paired with support groups (Rana et al. 2018) or peer mentors (Olusa et al. 2023). One study used multi‐component interventions including integration of agriculture, counseling, demonstrations, dialogue, and media, which showed improvements in minimum dietary diversity, minimum meal frequency, and minimum adequate diet. A second study included broad use of radio and television media messages and showed a significant increase in minimum dietary diversity and minimum adequate diet (Saaka et al. 2021).

#### 3.3.6 Randomized Trials

Fourteen trials included addressing young child nutrition (Effendy et al. 2020; Beatty et al. 2023; Tariku et al. 2015; Waswa et al. 2015; Kang et al. 2017; Han et al. 2019; Hewett et al. 2020). Several studies reported increased knowledge among the mothers following the intervention of community outreach (Siswati et al. 2022; Muluye et al. 2020), training and cooking demonstrations (Waswa et al. 2015), and/or receiving a booklet combined with group education (Mardani et al. 2024). These four studies also reported improved complementary feeding practices (Siswati et al. 2022; Muluye et al. 2020; Mardani et al. 2024) and minimum dietary diversity (Waswa et al. 2015). Few studies showed improved minimum dietary diversity and minimum mean frequency. Osaki et al. (2019) reported that the distribution of a Maternal and Child Health Handbook to eligible pregnant women who attend the health facility in Indonesia was effective in increasing both dietary diversity and minimum meal frequency among young children seen in clinic, although not exclusive breastfeeding and knowledge was mixed. Families kept the handbook at home and brought it with them to the health clinic allowing health facility staff to counsel families using the handbook and other job aids. Community outreach education varied in effectiveness with increasing meal frequency but not dietary diversity (Beatty et al. 2023; Tariku et al. 2015; Kang et al. 2017) or increasing dietary diversity but not meal frequency (Effendy et al. 2020; Han et al. 2019). The practice of providing food vouchers to participants was a useful intervention strategy that supported improving dietary diversity (Han et al. 2019). One study had a primary focus on education providers at the health facilities that led to an increase in both dietary diversity and meal frequency (Nikiema et al. 2017). A second intervention aimed at increasing use of the health facilities using mass media including television, billboards, and broadcasts. There were no significant differences between the groups and child feeding practices over time (Rawat et al. 2017).

In summary, the best intervention practice for increasing young child nutrition, specifically, complementary feeding practices and dietary diversity, is for trained community health workers to both educate families about best practices and provide cooking demonstrations to optimize nutrition. A second strategy is to enhance healthcare provider education along with family education. One example is to provide a booklet on young child nutrition to families and for the families to bring the booklet to the health facility for education and reinforcement of messages. Food vouchers were also effective in providing families the means of accessing a more diverse diet. The use of mass media education alone is not an effective intervention strategy.

#### 3.3.7 Antenatal Care and Maternal Nutrition

The primary outcomes reported for ANC and maternal nutrition interventions were accessing ANC care in the first trimester and at least four times during pregnancy and supplementation of iron and folic acid during pregnancy. The interventions included community outreach workers, media, and training healthcare providers at the facility.

#### 3.3.8 Nonrandomized Studies

There were only four studies that included ANC and maternal nutrition outcomes (Le Roux et al. 2020; Namukose et al. 2024; Dearden et al. 2023; Kung'u et al. 2018). Two studies used community outreach workers to provide counseling and support to mothers. In both cases, women who received the intervention had higher ANC visits at the health facility (Le Roux et al. 2020; Kung'u et al. 2018). In addition, Kung'u et al. (2018) also assessed and found higher consumption of iron and folic acid supplementation, facility‐based births, and facility‐based postnatal care. The same study found no intervention effect on attending one or more ANC visits and attending four or more ANC visits. In addition, there was no intervention effect on women consuming the recommended amount of iron and folic acid, nor early initiation of breastfeeding. Dearden et al. (2023) used media announcements, including further engagement of fathers in the pregnancy and child feeding practices with women reporting higher rates of attending four or more ANC visits compared with their previous pregnancy. Finally, an intervention focused on training healthcare workers in best practices found no significant difference in supplementation which they attributed to the healthcare facilities in general providing adequate supplementation to pregnant women (Namukose et al. 2024).

#### 3.3.9 Randomized Trials

Only three trials included ANC and maternal nutrition outcomes. Beatty et al. used health providers, including volunteers, midwives, and other clinic officials to provide village‐level counseling to pregnant and postpartum women. The intake of iron and folic acid supplementation was higher in the intervention condition; however, there was no difference in prenatal or postnatal checkups (Beatty et al. 2023). The intervention led by Lewycka et al. was a factorial design including women's groups, peer counseling, and usual care. Comparing women's groups to the usual care group revealed a 50% uptake in ANC and a 30% reduction in births attended by traditional birth attendants (Lewycka et al. 2013). Finally, Osaki et al. reported that the use of booklets, orientation, and job aids for healthcare staff increased the number of women who received more than six ANC visits (Osaki et al. 2019).

In summary, the best practices for increasing high‐quality ANC are mixed. Of the three high‐quality trials, each focused on a different aspect of the intervention. Training of healthcare providers and providing job aids along with community‐level counseling and women's groups by trained outreach workers should be tested in combination. Both interventions improved ANC practices, although the trials were tested independently. Adequate folic acid and iron supplementation was not effective in nonrandomized interventions; however, one hypothesis for this challenge was the lack of availability of the supplements themselves.

## 4 Discussion

Increasing best practices related to breastfeeding, young child nutrition, and ANC and maternal nutrition are priorities across many low‐resource contexts. Stunting, morbidity, and mortality are important indicators regionally, nationally, and globally. The United Nations Sustainable Development Goals (SDG) aim to: (1) end hunger, achieve food security and improved nutrition, and promote sustainable agriculture (SDG #2); and (2), ensure healthy lives and promote well‐being for all at all ages (SDG #3) (The United Nations Statistics Division n.d.). Many of the interventions presented in this review demonstrated an increase in knowledge and some healthful behaviors. Across the three broad areas of breastfeeding, young child nutrition, and ANC and maternal nutrition, the most promising practices were community outreach (Saaka et al. 2021; Rana et al. 2018; Fiorella et al. 2019; Kung'u et al. 2018; Tessema et al. 2023; Gebremariam et al. 2023; Kimani‐Murage et al. 2016; The United Nations Statistics Division n.d.), training at the facility level, and organizing social support through mothers' groups (Lewycka et al. 2013), grandmothers (Saaka et al. 2021; Fiorella et al. 2019; Tessema et al. 2023), or peer educators (Rana et al. 2018; Fiorella et al. 2019; Tessema et al. 2023). The involvement of fathers as a key component within these practices had mixed results (Saaka et al. 2021; Rana et al. 2018; Fiorella et al. 2019; Dearden et al. 2023; Tessema et al. 2023; Han et al. 2019; Gebremariam et al. 2023). Ultimately, this scoping review supports the conclusion that community‐based interventions are feasible, acceptable, and can shift usage of double‐duty actions.

The prevalence of breastfeeding in these contexts is high. In some cases, cultural beliefs that colostrum is not healthy limit early initiation of breastfeeding and prelacteal feeding, with this messaging coming from grandmothers or spouses and traditional birth attendants without training (Legesse et al. 2015; Rogers et al. 2011). Colostrum is the first milk a person's body produces during pregnancy and plays an important role in building a baby's immune system (World Health Organization 2009). Peer and other social support from trained community mentors provide a vital link to encourage the early initiation of breastfeeding. Most interventions in this review aimed at increasing the rate of exclusive breastfeeding for 6 months or more; however, Mbuya et al. (2019) found that while a higher proportion of women in the intervention compared to control groups exclusively breastfed for 6 months, exclusive breastfeeding declined for all women by 6 months. Possible explanations for this decline, include women returning to work and social pressure related to introducing complementary foods earlier. Further research should further articulate the rationale and identify community‐driven solutions to support women who breastfeed.

Young child feeding practices were very challenging to change. Dietary diversity, particularly in regions with poverty, low food security or food access, is limited in what foods are available. Most studies provided strategies for mothers to increase diversity using local food sources, alternative ways of preparing foods to optimize nutrition, and education about nutritional requirements for growth. The interventions were able to increase a food group or two, but the MDD total scores remained modest. Eshete et al. analyzed demographic and health survey data in Ethiopia and found that higher levels of paternal education, greater access to information (reading, listening to the radio), and higher income were associated with higher dietary diversity in children (Eshete et al. 2017). Breastfed children who met dietary diversity requirements were typically 6–11 months of age. A recent publication found that shared and equal decision making between spouses, participating in cooking demonstrations, and nutrition counseling through ANC visits were positively associated with greater dietary diversity in Ethiopia and are aligned with interventions to improve the double‐duty actions (Darebo et al. 2019).

Few studies focused on maternal nutrition and ANC. Maternal supplementation of folic acid reduces the risk of neural tube defects (US Preventive Services Task Force 2023) and iron supplementation can reduce the risk for premature or low‐birth weight infants and iron‐deficiency anemia (Nisar et al. 2020). Meeting the ANC recommended number and timing of visits and care by a trained provider monitors the mother's health, babies' growth, and provides opportunities for counseling the mother on best practices and warning signs. Although this scoping review did not find many interventions focused specifically on this topic, many studies did initiate while the woman was pregnant, allowing some key counseling messages to be shared. Adolescents should be made aware of the value of early ANC and maternal nutrition prior to pregnancy. This may support greater use of ANC. Strategies need to be developed to address the unique challenges of women living in rural areas as they navigate long distances and costs associated with accessing care at a health facility.

The concepts of sustainability and scalability are related yet distinct concepts. Sustainability is a multidimensional term that at its core refers to the continuation of programming (Shediac‐Rizkallah and Bone 1998). Community capacity building is one strategy for sustainability, as broad behavioral change, such as exclusive breastfeeding or early initiation of breastfeeding, can change community norms of what is acceptable culturally and thus sustain the feeding practices (Shediac‐Rizkallah and Bone 1998). The best practices identified in this scoping review support the idea of sustainability because of the training of community members to be a source of health information and those community members are linked to the healthcare institutions (Shediac‐Rizkallah and Bone 1998). Yet, sustainability may be viewed as a spontaneous occurrence as opposed to an intentional process including the expansion of reach, formal transfer of control to local implementers, and “retaining the effectiveness” of the program (Zamboni et al. 2019). The behavioral interventions in this review demonstrated feasibility yet little consideration was placed on planning for scalability. It is vital for complex interventions to not only plan for sustainability and scalability, but also to consider the “socio‐political context and health system” structures in which they are being implemented (Zamboni et al. 2019). For example, Ethiopia incorporates community health workers within their public health system to conduct community‐based health activities and education (Ministry of Health 2023). At the same time, Ethiopia is a complex country with 12 regional states, 2 chartered cities, over 100 languages, over 80 distinct cultural groups, and urban and rural populations (Mehretu et al. 2023). Sustainability and scalability will require national commitment for ongoing training, resources, and cultural/linguistic adaptation.

## 5 Limitations

This scoping review excluded studies in which the primary intervention included fortified food products to increase minimum dietary diversity. Food fortification is important and not universally available. It is likely that if those studies were included, our conclusions about MDD impacts would have been more positive, yet the use of local food knowledge to enhance micronutrients and dietary intake is more sustainable. Despite protocols, it is possible there was bias or error in screening or in extraction or there was publication bias. These situations would limit the generalizability of our findings and likely have led to an overstating of what is effective. This scoping review did not specifically report on sanitation and did not focus on the impact of the interventions on child growth. There is strong evidence that the evidence‐based interventions are important for child growth. The range of articles included has varying degrees of “dose” of the intervention that restricted clear cross‐study analysis. Future research should include clear evidence of the dose–response relationship of interventions and growth outcomes. Finally, not all countries in sub‐Saharan Africa and SE Asia had studies represented in the literature and large countries had interventions in only small regions of the country. Therefore, it is possible that there is both publication bias and within‐country intervention bias, limiting the generalizability.

## 6 Conclusions

This scoping review highlighted the importance of tailoring messages to a specific context while implementing evidence‐based approaches. This is particularly vital for introducing complementary foods and young child nutrition as the diet must be tailored to what is available. Building peer and community social support based on key and evidence‐based messaging provides an important opportunity to improve the lives of women, children, and the next generation. Given that studies were drawn from a variety of countries from sub‐Saharan Africa and SE Asia, the findings from this review have broad implications for similar countries in these regions. In addition, the scale of implementation for the interventions used in the included studies varies—future research should examine both the spectrum of feasibility and efficacy across wide‐scale and small‐scale interventions.

## Author Contributions

**Mary O. Hearst:** conceptualization (equal), data curation (equal), formal analysis (equal), investigation (equal), methodology (equal), project administration (equal), writing – original draft (equal), writing – review and editing (equal). **Elizabeth Weinfurter:** data curation (equal), methodology (equal). **Lillian Norman:** data curation (supporting), formal analysis (equal). **Clara A. Normile:** data curation (equal), validation (equal). **Hussen Mekonnen:** writing – review and editing (equal). **Melissa N. Laska:** conceptualization (equal), writing – review and editing (equal).

## Ethics Statement

The authors have nothing to report.

## Consent

The authors have nothing to report.

## Conflicts of Interest

The authors declare no conflicts of interest.

## Data Availability

The final protocol is registered with the Open Science Framework.

## Appendix 1

### Search Strategies

Simplified strategy:

**Table:**

1. (child* or p?ediatric* or infant* or toddler*).ti,ab.	330317
2. (malnutrition or malnourish* or undernutrition or undernourish* or (nutrition adj1 disorder*)).mp.	53082
3. (stunt* or wasting or wasted or anthropometric failure* or (growth adj1 (disorder* or retard*))).mp.	53153
4. (overweight or overnutrition or hypernutrition or obes*).mp.	177693
5. (dual burden or double burden).mp.	1145
6. or/2‐5	271296
7. 1 and 6	55995
8. (intervention* or program* or trial* or experiment* or policy or policies).mp.	2884287
9. (breast fe* or breastfe*).mp.	35187
10. (complementary adj3 (food* or feed* or fed)).mp.	4630
11. ((care or educat*) adj3 (antenatal* or ante‐natal* or prenatal* or pre‐natal*)).mp.	11272
12. 9 or 10 or 11	47844
13. [geography search hedge]	888847
14. 7 and 8 and 12 and 13	853
15. limit 14 to (english language and yr=“2010 ‐Current”)	700

### Medline via Ovid Search Strategy

Ovid MEDLINE(R) ALL <1946 to October 22, 2024>

Search link

**Table:**

1. exp Nutrition Disorders/ or (malnutrition or malnourish* or undernutrition or undernourish*).mp. or (nutrition adj1 disorder*).mp.	481616
2. exp Growth Disorders/ or (stunt* or wasting or wasted or anthropometric failure*).mp. or (growth adj1 (disorder* or retard*)).mp.	92677
3. exp Overnutrition/ or (overweight or overnutrition or hypernutrition or obes*).mp.	498534
4. (dual burden or double burden).mp.	1771
5. 1 or 2 or 3 or 4	765457
6. (child* or p?ediatric* or infant* or toddler*).ti,ab.	2261397
7. 5 and 6	131628
8. exp Breast Feeding/ or (breast fe* or breastfe*).mp.	72803
9. (complementary adj3 (food* or feed* or fed)).mp.	4247
10. exp Prenatal Care/ or ((care or educat*) adj3 (antenatal* or ante‐natal* or prenatal* or pre‐natal*)).mp.	52600
11. 8 or 9 or 10	124611
12. (intervention* or program* or trial* or experiment* or policy or policies).mp.	7326615
13. exp Africa, Eastern/ or (east* adj2 africa*).mp. or British Indian Ocean Territory.mp. or Burundi*.mp. or Comoros.mp. or Djibouti*.mp. or Eritrea*.mp. or Ethiopia*.mp. or Kenya*.mp. or Madagascar.mp. or Malawi.mp. or Mauritius.mp. or Mayotte.mp. or Mozambique.mp. or Reunion.mp. or Rwanda*.mp. or Seychelles.mp. or Somalia*.mp. or Sudan*.mp. or Tanzania*.mp. or Uganda*.mp. or Zambia.mp. or Zimbabwe.mp. or Crozet Islands.mp. or Iles Crozet.mp. or Scattered Islands.mp. or Iles Eparses.mp. or Addis Ababa.mp. or Asmara.mp. or Anananarivo.mp. or Arusha.mp. or Axum.mp. or Bahir Dar.mp. or Berbera.mp. or Bulawayo.mp. or Dese.mp. or Eldoret.mp. or Garissa.mp. or Geita.mp. or Gondar.mp. or Great Rift Valley.mp. or Hargeisa.mp. or Hargeysa.mp. or Hola.mp. or Jinja.mp. or Iringa.mp. or Kigoma.mp. or Jimma.mp. or Korogwe.mp. or Nairobi.mp. or “Dar es Salaam”.mp. or Mombasa.mp. or Mogadishu.mp. or Dodomoa.mp. or Bujumbura.mp. or Mbeya.mp. or Lusaka.mp. or Harare.mp. or Kakamega.mp. or Kampala.mp. or Kigali.mp. or Kire Dawa.mp. or Kikuyu.mp. or Kisumu.mp. or Kitale.mp. or Kitui.mp. or Lilongwe.mp. or Lake Victoria.mp. or Lake Tanganyika.mp. or Lamu.mp. or Lodwar.mp. or Lokichogio.mp. or Malindi.mp. or Machakos.mp. or Marka.mp. or Machakos.mp. or Maputo.mp. or Maralal.mp. or Mek'ele.mp. or Meru.mp. or Musoma.mp. or Mtwara.mp. or Mumias.mp. or Moshi.mp. or Moroni.mp. or Morogoro.mp. or Mwanza.mp. or Naivasha.mp. or Nanyuki.mp. or Nakuru.mp. or Namanga.mp. or Nyeri.mp. or Port Louis.mp. or Puntland*.mp. or Nyahururu.mp. or Kismayo.mp. or Ruiru.mp. or Rwenzori Mountains.mp. or Sinyanga.mp. or Songea.mp. or tanga.mp. or Tabora.mp. or voi.mp. or webuye.mp. or Zanzibar.mp. or ((Adiharush or Ali‐Addeh or Alinjugur or Buramino or Dadaab or Dagahaley or Dollo Ado or Fugnido or Hagadera or Hilaweyn or Ifo or Kakuma or Kambioos or Kayaka II or Kobe or Kyangwali or Nakivale or Nyarugusu or Wad Sherife or Bokolmanyo or Melkadida or Rwamanja) adj5 (camp or refug*)).ti,ab. or exp africa, western/ or ((africa* adj2 west*) or benin* or burkina fas* or cape verd* or cabo verd* or ivory coast or “cote d'ivoire*” or gambia* or ghana* or guinea* or bissau or liberia* or (mali not fowl) or malian or mauritania* or nigeria* or senegal* or sierra leon* or togo*).mp. or (Lagos or Accra or Abidjan or Dakar or Abobo or Abuja or Freetown or Ouagadougou or Conakry or Lome or Bamako or Cotonou or Kumasi or Monrovia or Ibadan or Kano or Port Harcourt or Benin City or Porto Novo or Niamey or Yamoussoukro or Banjul or Timbuktu or Djenne or Abomeyu or Zaria or Tamale or Jos or Cape Coast or Maidugul or Aba or Gao or Calabar or Warri or Maiduguri or Bobo Dioulasso or Parakou or Djougou or Bohicon or Sekondi Takoradi or Sunyani or Obuasi or Teshie or Tema or Sikasso or Kalabankoro or Nouakchott or Dakhlet Nouadhibou or Benin City or Port Harcourt or Ilorin or Kaduna or Enugu or Ikorodu or Onitsha or Bauchi or Akure or Abeokuta or Sokoto or Bouake or Makeni or Kaduan or Sosgbo or Osogbo or Gombe or Ilesa or Badagry or makurdi or Sagamu or Iseyin or obbomosho or Awka or Ado Ekiti or Nsukka or Ikeja or Katsina or Okene or Lafia or Minna or Ondo city or Umuahia or Calabar or Yola or Pikine or Touba or “Thies Nones” or Saint Louis or Kolak or Ziguinch or (San Pedro not (Spain or Mexico or Argentina or California or United States or Italy)) or Bandama or Daloa or Owerri or Kandi or Ifi or Dakar or Ogbomosho or Divo or Korhogo).ti,ab. or exp africa, central/ or ((africa adj2 central) or angola or cameroon* or chad or tchad or congo* or DRC or equatorial guinea* or gabon* or Sao Tome or Principe or Luanda or lobito or kuito or huambo or Malanje or Douala or Yaounde or Bamenda or Garoua of Bafoussam or Nganoundere or Maroua or Kouosseri or Buena or Kumba or “N'Djamena” or Moundou or Bangui or Bimbo or Brazzaville or Point Noire or Kinshasa or Lubumbashi or Leopoldville or Elizabethville or Mbuji Mayi or Bakwanga or Bukavu or Costermansville or Kananga or Luluabourg or Kisangani or Stanleyville or Tshikapa or Koalwezi or Likasi or Jadotville or Goma or Kikwit or Uvira or Bunia or Mbandaka or Coquilhatville or Matadi or Butembo or Kabinda or Mwene Ditu or Isiro or Paulis or Boma or Kindu or Bata or Malabo or Libreville).ti,ab. or exp africa, southern/ or ((africa* adj2 south*) or angola* or botswana* or lesotho* or malawi* or mozambiq* or namibia* or swaziland or zambia* or zimbabwe or Zulu or Tsonga or Xhosa or Swazi or Ndebele or Tswana or Sotho or Shona people or BaLunda or Mbundu or Ovimbundu or Chaga or Sukuma or Pretoria or Cape Town or Johannesburg or Durban or Port Elizabeth or Bloemfontein or Windhoek or Maseru or Pietermaritz or (Kimberley not Australia) or Nespruit or Soweto or Polokwane or Limpopo or Rustenburg or Mahikeng or Oudtshroom or Stellenbosch or Paarl or Gaborone or Luanda or Cabinda or Huambo or Lubango or Kuit or Malanje or Lobito or Lilongwe or Blantyre or Mzuzu or Maputo or Matola or Beira or Nampula or Chimoio or Nacala or Quelimane or Lusaka or Kitwe or Ndola or Kabwe or Copperbelt or Harare or Bulawayo or Chitungwiza or Mutare or Masvingo or Monashonaland or Manicaland).ti,ab. or exp “Africa South of the Sahara”/ or exp Asia, Southeastern/ or (“sub sahara*” or subsahara* or “south of the sahara”).mp. or (Indonesia or Vietnam* or Laos or Brunei or Thailand or Myanmar or Philippines or Cambodia or Singapore or Malaysia or southeast* asia).mp.	798453
14. 7 and 11 and 12 and 13	1054
15. limit 14 to (english language and yr=”2010 ‐Current”)	822

### Agricola via Ovid Search Strategy

AGRICOLA <1970 to October 2024>

Search link

**Table:**

1. (child* or p?ediatric* or infant* or toddler*).ti,ab.	102526
2. exp nutrition/ or exp malnutrition/ or (malnutrition or malnourish* or undernutrition or undernourish*).mp. or (nutrition adj1 disorder*).mp.	612030
3. exp growth retardation/or (stunt* or wasting or wasted or anthropometric failure*).mp. or (growth adj1 (disorder* or retard*)).mp.	31886
4. exp overweight/ or (overweight or overnutrition or hypernutrition or obes*).mp.	64727
5. (dual burden or double burden).mp.	304
6. or/2‐5	667516
7. 1 and 6	45676
8. (intervention* or program* or trial* or experiment* or policy or policies).mp.	1385313
9. exp breast feeding/ or (breast fe* or breastfe*).mp.	8323
10. (complementary adj3 (food* or feed* or fed)).mp.	1581
11. exp prenatal care/ or ((care or educat*) adj3 (antenatal* or ante‐natal* or prenatal* or pre‐natal*)).mp.	1174
12. 9 or 10 or 11	10408
13. exp Africa, Eastern/ or (east* adj2 africa*).mp. or British Indian Ocean Territory.mp. or Burundi*.mp. or Comoros.mp. or Djibouti*.mp. or Eritrea*.mp. or Ethiopia*.mp. or Kenya*.mp. or Madagascar.mp. or Malawi.mp. or Mauritius.mp. or Mayotte.mp. or Mozambique.mp. or Reunion.mp. or Rwanda*.mp. or Seychelles.mp. or Somalia*.mp. or Sudan*.mp. or Tanzania*.mp. or Uganda*.mp. or Zambia.mp. or Zimbabwe.mp. or Crozet Islands.mp. or Iles Crozet.mp. or Scattered Islands.mp. or Iles Eparses.mp. or Addis Ababa.mp. or Asmara.mp. or Anananarivo.mp. or Arusha.mp. or Axum.mp. or Bahir Dar.mp. or Berbera.mp. or Bulawayo.mp. or Dese.mp. or Eldoret.mp. or Garissa.mp. or Geita.mp. or Gondar.mp. or Great Rift Valley.mp. or Hargeisa.mp. or Hargeysa.mp. or Hola.mp. or Jinja.mp. or Iringa.mp. or Kigoma.mp. or Jimma.mp. or Korogwe.mp. or Nairobi.mp. or “Dar es Salaam”.mp. or Mombasa.mp. or Mogadishu.mp. or Dodomoa.mp. or Bujumbura.mp. or Mbeya.mp. or Lusaka.mp. or Harare.mp. or Kakamega.mp. or Kampala.mp. or Kigali.mp. or Kire Dawa.mp. or Kikuyu.mp. or Kisumu.mp. or Kitale.mp. or Kitui.mp. or Lilongwe.mp. or Lake Victoria.mp. or Lake Tanganyika.mp. or Lamu.mp. or Lodwar.mp. or Lokichogio.mp. or Malindi.mp. or Machakos.mp. or Marka.mp. or Machakos.mp. or Maputo.mp. or Maralal.mp. or Mek'ele.mp. or Meru.mp. or Musoma.mp. or Mtwara.mp. or Mumias.mp. or Moshi.mp. or Moroni.mp. or Morogoro.mp. or Mwanza.mp. or Naivasha.mp. or Nanyuki.mp. or Nakuru.mp. or Namanga.mp. or Nyeri.mp. or Port Louis.mp. or Puntland*.mp. or Nyahururu.mp. or Kismayo.mp. or Ruiru.mp. or Rwenzori Mountains.mp. or Sinyanga.mp. or Songea.mp. or tanga.mp. or Tabora.mp. or voi.mp. or webuye.mp. or Zanzibar.mp. or ((Adiharush or Ali‐Addeh or Alinjugur or Buramino or Dadaab or Dagahaley or Dollo Ado or Fugnido or Hagadera or Hilaweyn or Ifo or Kakuma or Kambioos or Kayaka II or Kobe or Kyangwali or Nakivale or Nyarugusu or Wad Sherife or Bokolmanyo or Melkadida or Rwamanja) adj5 (camp or refug*)).ti,ab. or exp africa, western/ or ((africa* adj2 west*) or benin* or burkina fas* or cape verd* or cabo verd* or ivory coast or “cote d'ivoire*” or gambia* or ghana* or guinea* or bissau or liberia* or (mali not fowl) or malian or mauritania* or nigeria* or senegal* or sierra leon* or togo*).mp. or (Lagos or Accra or Abidjan or Dakar or Abobo or Abuja or Freetown or Ouagadougou or Conakry or Lome or Bamako or Cotonou or Kumasi or Monrovia or Ibadan or Kano or Port Harcourt or Benin City or Porto Novo or Niamey or Yamoussoukro or Banjul or Timbuktu or Djenne or Abomeyu or Zaria or Tamale or Jos or Cape Coast or Maidugul or Aba or Gao or Calabar or Warri or Maiduguri or Bobo Dioulasso or Parakou or Djougou or Bohicon or Sekondi Takoradi or Sunyani or Obuasi or Teshie or Tema or Sikasso or Kalabankoro or Nouakchott or Dakhlet Nouadhibou or Benin City or Port Harcourt or Ilorin or Kaduna or Enugu or Ikorodu or Onitsha or Bauchi or Akure or Abeokuta or Sokoto or Bouake or Makeni or Kaduan or Sosgbo or Osogbo or Gombe or Ilesa or Badagry or makurdi or Sagamu or Iseyin or obbomosho or Awka or Ado Ekiti or Nsukka or Ikeja or Katsina or Okene or Lafia or Minna or Ondo city or Umuahia or Calabar or Yola or Pikine or Touba or “Thies Nones” or Saint Louis or Kolak or Ziguinch or (San Pedro not (Spain or Mexico or Argentina or California or United States or Italy)) or Bandama or Daloa or Owerri or Kandi or Ifi or Dakar or Ogbomosho or Divo or Korhogo).ti,ab. or exp africa, central/ or ((africa adj2 central) or angola or cameroon* or chad or tchad or congo* or DRC or equatorial guinea* or gabon* or Sao Tome or Principe or Luanda or lobito or kuito or huambo or Malanje or Douala or Yaounde or Bamenda or Garoua of Bafoussam or Nganoundere or Maroua or Kouosseri or Buena or Kumba or “N'Djamena” or Moundou or Bangui or Bimbo or Brazzaville or Point Noire or Kinshasa or Lubumbashi or Leopoldville or Elizabethville or Mbuji Mayi or Bakwanga or Bukavu or Costermansville or Kananga or Luluabourg or Kisangani or Stanleyville or Tshikapa or Koalwezi or Likasi or Jadotville or Goma or Kikwit or Uvira or Bunia or Mbandaka or Coquilhatville or Matadi or Butembo or Kabinda or Mwene Ditu or Isiro or Paulis or Boma or Kindu or Bata or Malabo or Libreville).ti,ab. or exp africa, southern/ or ((africa* adj2 south*) or angola* or botswana* or lesotho* or malawi* or mozambiq* or namibia* or swaziland or zambia* or zimbabwe or Zulu or Tsonga or Xhosa or Swazi or Ndebele or Tswana or Sotho or Shona people or BaLunda or Mbundu or Ovimbundu or Chaga or Sukuma or Pretoria or Cape Town or Johannesburg or Durban or Port Elizabeth or Bloemfontein or Windhoek or Maseru or Pietermaritz or (Kimberley not Australia) or Nespruit or Soweto or Polokwane or Limpopo or Rustenburg or Mahikeng or Oudtshroom or Stellenbosch or Paarl or Gaborone or Luanda or Cabinda or Huambo or Lubango or Kuit or Malanje or Lobito or Lilongwe or Blantyre or Mzuzu or Maputo or Matola or Beira or Nampula or Chimoio or Nacala or Quelimane or Lusaka or Kitwe or Ndola or Kabwe or Copperbelt or Harare or Bulawayo or Chitungwiza or Mutare or Masvingo or Monashonaland or Manicaland).ti,ab. or (“sub sahara*” or subsahara* or “south of the sahara”).mp. or exp South East Asia/ or (Indonesia or Vietnam* or Laos or Brunei or Thailand or Myanmar or Philippines or Cambodia or Singapore or Malaysia or southeast* asia or south east asia).mp.	275492
14. 7 and 8 and 12 and 13	564
15. limit 14 to (english language and yr=“2010 ‐Current”)	429

### CAB Abstracts via Ovid Search Strategy

CAB Abstracts <1973 to 2024 Week 42>

Search link

**Table:**

1. (child* or p?ediatric* or infant* or toddler*).ti,ab.	330317
2. (malnutrition or malnourish* or undernutrition or undernourish* or (nutrition adj1 disorder*)).mp.	53082
3. (stunt* or wasting or wasted or anthropometric failure* or (growth adj1 (disorder* or retard*))).mp.	53153
4. (overweight or overnutrition or hypernutrition or obes*).mp.	177693
5. (dual burden or double burden).mp.	1145
6. or/2‐5	271296
7. 1 and 6	55995
8. (intervention* or program* or trial* or experiment* or policy or policies).mp.	2884287
9. (breast fe* or breastfe*).mp.	35187
10. (complementary adj3 (food* or feed* or fed)).mp.	4630
11. ((care or educat*) adj3 (antenatal* or ante‐natal* or prenatal* or pre‐natal*)).mp.	11272
12. 9 or 10 or 11	47844
13. ((east* adj2 africa*) or British Indian Ocean Territory or Burundi* or Comoros or Djibouti* or Eritrea* or Ethiopia* or Kenya* or Madagascar or Malawi or Mauritius or Mayotte or Mozambique or Reunion or Rwanda* or Seychelles or Somalia* or Sudan* or Tanzania* or Uganda* or Zambia or Zimbabwe or Crozet Islands or Iles Crozet or Scattered Islands or Iles Eparses or Addis Ababa or Asmara or Anananarivo or Arusha or Axum or Bahir Dar or Berbera or Bulawayo or Dese or Eldoret or Garissa or Geita or Gondar or Great Rift Valley or Hargeisa or Hargeysa or Hola or Jinja or Iringa or Kigoma or Jimma or Korogwe or Nairobi or “Dar es Salaam” or Mombasa or Mogadishu or Dodomoa or Bujumbura or Mbeya or Lusaka or Harare or Kakamega or Kampala or Kigali or Kire Dawa or Kikuyu or Kisumu or Kitale or Kitui or Lilongwe or Lake Victoria or Lake Tanganyika or Lamu or Lodwar or Lokichogio or Malindi or Machakos or Marka or Machakos or Maputo or Maralal or Mek'ele or Meru or Musoma or Mtwara or Mumias or Moshi or Moroni or Morogoro or Mwanza or Naivasha or Nanyuki or Nakuru or Namanga or Nyeri or Port Louis or Puntland* or Nyahururu or Kismayo or Ruiru or Rwenzori Mountains or Sinyanga or Songea or tanga or Tabora or voi or webuye or Zanzibar).mp. or ((Adiharush or Ali‐Addeh or Alinjugur or Buramino or Dadaab or Dagahaley or Dollo Ado or Fugnido or Hagadera or Hilaweyn or Ifo or Kakuma or Kambioos or Kayaka II or Kobe or Kyangwali or Nakivale or Nyarugusu or Wad Sherife or Bokolmanyo or Melkadida or Rwamanja) adj5 (camp or refug*)).ti,ab. or ((africa* adj2 west*) or benin* or burkina fas* or cape verd* or cabo verd* or ivory coast or “cote d'ivoire*” or gambia* or ghana* or guinea* or bissau or liberia* or (mali not fowl) or malian or mauritania* or nigeria* or senegal* or sierra leon* or togo*).mp. or (Lagos or Accra or Abidjan or Dakar or Abobo or Abuja or Freetown or Ouagadougou or Conakry or Lome or Bamako or Cotonou or Kumasi or Monrovia or Ibadan or Kano or Port Harcourt or Benin City or Porto Novo or Niamey or Yamoussoukro or Banjul or Timbuktu or Djenne or Abomeyu or Zaria or Tamale or Jos or Cape Coast or Maidugul or Aba or Gao or Calabar or Warri or Maiduguri or Bobo Dioulasso or Parakou or Djougou or Bohicon or Sekondi Takoradi or Sunyani or Obuasi or Teshie or Tema or Sikasso or Kalabankoro or Nouakchott or Dakhlet Nouadhibou or Benin City or Port Harcourt or Ilorin or Kaduna or Enugu or Ikorodu or Onitsha or Bauchi or Akure or Abeokuta or Sokoto or Bouake or Makeni or Kaduan or Sosgbo or Osogbo or Gombe or Ilesa or Badagry or makurdi or Sagamu or Iseyin or obbomosho or Awka or Ado Ekiti or Nsukka or Ikeja or Katsina or Okene or Lafia or Minna or Ondo city or Umuahia or Calabar or Yola or Pikine or Touba or “Thies Nones” or Saint Louis or Kolak or Ziguinch or (San Pedro not (Spain or Mexico or Argentina or California or United States or Italy)) or Bandama or Daloa or Owerri or Kandi or Ifi or Dakar or Ogbomosho or Divo or Korhogo).ti,ab. or ((africa adj2 central) or angola or cameroon* or chad or tchad or congo* or DRC or equatorial guinea* or gabon* or Sao Tome or Principe or Luanda or lobito or kuito or huambo or Malanje or Douala or Yaounde or Bamenda or Garoua of Bafoussam or Nganoundere or Maroua or Kouosseri or Buena or Kumba or “N'Djamena” or Moundou or Bangui or Bimbo or Brazzaville or Point Noire or Kinshasa or Lubumbashi or Leopoldville or Elizabethville or Mbuji Mayi or Bakwanga or Bukavu or Costermansville or Kananga or Luluabourg or Kisangani or Stanleyville or Tshikapa or Koalwezi or Likasi or Jadotville or Goma or Kikwit or Uvira or Bunia or Mbandaka or Coquilhatville or Matadi or Butembo or Kabinda or Mwene Ditu or Isiro or Paulis or Boma or Kindu or Bata or Malabo or Libreville).ti,ab. or ((africa* adj2 south*) or angola* or botswana* or lesotho* or malawi* or mozambiq* or namibia* or swaziland or zambia* or zimbabwe or Zulu or Tsonga or Xhosa or Swazi or Ndebele or Tswana or Sotho or Shona people or BaLunda or Mbundu or Ovimbundu or Chaga or Sukuma or Pretoria or Cape Town or Johannesburg or Durban or Port Elizabeth or Bloemfontein or Windhoek or Maseru or Pietermaritz or (Kimberley not Australia) or Nespruit or Soweto or Polokwane or Limpopo or Rustenburg or Mahikeng or Oudtshroom or Stellenbosch or Paarl or Gaborone or Luanda or Cabinda or Huambo or Lubango or Kuit or Malanje or Lobito or Lilongwe or Blantyre or Mzuzu or Maputo or Matola or Beira or Nampula or Chimoio or Nacala or Quelimane or Lusaka or Kitwe or Ndola or Kabwe or Copperbelt or Harare or Bulawayo or Chitungwiza or Mutare or Masvingo or Monashonaland or Manicaland).ti,ab. or (“sub sahara*” or subsahara* or “south of the sahara”).mp. or (Indonesia or Vietnam* or Laos or Brunei or Thailand or Myanmar or Philippines or Cambodia or Singapore or Malaysia or southeast* asia or south east asia).mp.	888847
14. 7 and 8 and 12 and 13	853
15. limit 14 to (english language and yr=“2010 ‐Current”)	700

### Scopus Search Strategy

Search link

((TITLE‐ABS‐KEY(malnutrition OR malnourish* OR undernutrition OR undernourish* OR (nutrition W/1 disorder* )) OR TITLE‐ABS‐KEY(stunt* OR wasting OR wasted OR “anthropometric failure*” OR (growth W/1 (disorder* OR retard* ))) OR TITLE‐ABS‐KEY(overweight OR overnutrition OR hypernutrition OR obes* ) OR TITLE‐ABS‐KEY(“dual burden” OR “double burden” )) AND (TITLE‐ABS(child* OR pediatric* OR paediatric* OR infant* OR toddler* )) AND TITLE‐ABS‐KEY(“breast fe*” OR breastfe* ) OR TITLE‐ABS‐KEY(complementary W/3 (food* OR feed* OR fed )) OR TITLE‐ABS‐KEY((care OR educat* ) W/3 (antenatal* OR ante‐natal* OR prenatal* OR pre‐natal* )) AND TITLE‐ABS‐KEY(intervention* OR program* OR trial* OR experiment* OR policy OR policies )) AND (TITLE‐ABS‐KEY((east* W/2 africa* ) OR “British Indian Ocean Territory” OR Burundi* OR Comoros OR Djibouti* OR Eritrea* OR Ethiopia* OR Kenya* OR Madagascar OR Malawi OR Mauritius OR Mayotte OR Mozambique OR Reunion OR Rwanda* OR Seychelles OR Somalia* OR Sudan* OR Tanzania* OR Uganda* OR Zambia OR Zimbabwe OR “Crozet Islands” OR “Iles Crozet” OR “Scattered Islands” OR “Iles Eparses” OR “Addis Ababa” OR Asmara OR Anananarivo OR Arusha OR Axum OR “Bahir Dar” OR Berbera OR Bulawayo OR Dese OR Eldoret OR Garissa OR Geita OR Gondar OR “Great Rift Valley” OR Hargeisa OR Hargeysa OR Hola OR Jinja OR Iringa OR Kigoma OR Jimma OR Korogwe OR Nairobi OR “Dar es Salaam” OR Mombasa OR Mogadishu OR Dodomoa OR Bujumbura OR Mbeya OR Lusaka OR Harare OR Kakamega OR Kampala OR Kigali OR “Kire Dawa” OR Kikuyu OR Kisumu OR Kitale OR Kitui OR Lilongwe OR “Lake Victoria” OR “Lake Tanganyika” OR Lamu OR Lodwar OR Lokichogio OR Malindi OR Machakos OR Marka OR Machakos OR Maputo OR Maralal OR Mek'ele OR Meru OR Musoma OR Mtwara OR Mumias OR Moshi OR Moroni OR Morogoro OR Mwanza OR Naivasha OR Nanyuki OR Nakuru OR Namanga OR Nyeri OR “Port Louis” OR Puntland* OR Nyahururu OR Kismayo OR Ruiru OR “Rwenzori Mountains” OR Sinyanga OR Songea OR tanga OR Tabora OR voi OR webuye OR Zanzibar ) OR TITLE‐ABS((Adiharush OR Ali‐Addeh OR Alinjugur OR Buramino OR Dadaab OR Dagahaley OR “Dollo Ado” OR Fugnido OR Hagadera OR Hilaweyn OR Ifo OR Kakuma OR Kambioos OR “Kayaka II” OR Kobe OR Kyangwali OR Nakivale OR Nyarugusu OR “Wad Sherife” OR Bokolmanyo OR Melkadida OR Rwamanja ) W/5 (camp OR refug* )) OR TITLE‐ABS‐KEY((africa* W/2 west* ) OR benin* OR “burkina fas*” OR “cape verd*” OR “cabo verd*” OR “ivory coast” OR “cote d'ivoire*” OR gambia* OR ghana* OR guinea* OR bissau OR liberia* OR (mali NOT fowl ) OR malian OR mauritania* OR nigeria* OR senegal* OR “sierra leon*” OR togo* ) OR TITLE‐ABS(Lagos OR Accra OR Abidjan OR Dakar OR Abobo OR Abuja OR Freetown OR Ouagadougou OR Conakry OR Lome OR Bamako OR Cotonou OR Kumasi OR Monrovia OR Ibadan OR Kano OR “Port Harcourt” OR “Benin City” OR “Porto Novo” OR Niamey OR Yamoussoukro OR Banjul OR Timbuktu OR Djenne OR Abomeyu OR Zaria OR Tamale OR Jos OR “Cape Coast” OR Maidugul OR Aba OR Gao OR Calabar OR Warri OR Maiduguri OR “Bobo Dioulasso” OR Parakou OR Djougou OR Bohicon OR “Sekondi Takoradi” OR Sunyani OR Obuasi OR Teshie OR Tema OR Sikasso OR Kalabankoro OR Nouakchott OR “Dakhlet Nouadhibou” OR “Benin City” OR “Port Harcourt” OR Ilorin OR Kaduna OR Enugu OR Ikorodu OR Onitsha OR Bauchi OR Akure OR Abeokuta OR Sokoto OR Bouake OR Makeni OR Kaduan OR Sosgbo OR Osogbo OR Gombe OR Ilesa OR Badagry OR makurdi OR Sagamu OR Iseyin OR obbomosho OR Awka OR “Ado Ekiti” OR Nsukka OR Ikeja OR Katsina OR Okene OR Lafia OR Minna OR “Ondo city” OR Umuahia OR Calabar OR Yola OR Pikine OR Touba OR “Thies Nones” OR “Saint Louis” OR Kolak OR Ziguinch OR Bandama OR Daloa OR Owerri OR Kandi OR Ifi OR Dakar OR Ogbomosho OR Divo OR Korhogo ) OR TITLE‐ABS((africa W/2 central ) OR angola OR cameroon* OR chad OR tchad OR congo* OR DRC OR “equatorial guinea*” OR gabon* OR “Sao Tome” OR Principe OR Luanda OR lobito OR kuito OR huambo OR Malanje OR Douala OR Yaounde OR Bamenda OR “Garoua of Bafoussam” OR Nganoundere OR Maroua OR Kouosseri OR Buena OR Kumba OR N'Djamena OR Moundou OR Bangui OR Bimbo OR Brazzaville OR “Point Noire” OR Kinshasa OR Lubumbashi OR Leopoldville OR Elizabethville OR “Mbuji Mayi” OR Bakwanga OR Bukavu OR Costermansville OR Kananga OR Luluabourg OR Kisangani OR Stanleyville OR Tshikapa OR Koalwezi OR Likasi OR Jadotville OR Goma OR Kikwit OR Uvira OR Bunia OR Mbandaka OR Coquilhatville OR Matadi OR Butembo OR Kabinda OR “Mwene Ditu” OR Isiro OR Paulis OR Boma OR Kindu OR Bata OR Malabo OR Libreville ) OR TITLE‐ABS((africa* W/2 south* ) OR angola* OR botswana* OR lesotho* OR malawi* OR mozambiq* OR namibia* OR swaziland OR zambia* OR zimbabwe OR Zulu OR Tsonga OR Xhosa OR Swazi OR Ndebele OR Tswana OR Sotho OR “Shona people” OR BaLunda OR Mbundu OR Ovimbundu OR Chaga OR Sukuma OR Pretoria OR “Cape Town” OR Johannesburg OR Durban OR “Port Elizabeth” OR Bloemfontein OR Windhoek OR Maseru OR Pietermaritz OR (Kimberley NOT Australia ) OR Nespruit OR Soweto OR Polokwane OR Limpopo OR Rustenburg OR Mahikeng OR Oudtshroom OR Stellenbosch OR Paarl OR Gaborone OR Luanda OR Cabinda OR Huambo OR Lubango OR Kuit OR Malanje OR Lobito OR Lilongwe OR Blantyre OR Mzuzu OR Maputo OR Matola OR Beira OR Nampula OR Chimoio OR Nacala OR Quelimane OR Lusaka OR Kitwe OR Ndola OR Kabwe OR Copperbelt OR Harare OR Bulawayo OR Chitungwiza OR Mutare OR Masvingo OR Monashonaland OR Manicaland ) OR TITLE‐ABS‐KEY(“sub sahara*” OR subsahara* OR “south of the sahara” ) OR TITLE‐ABS‐KEY(Indonesia OR Vietnam* OR Laos OR Brunei OR Thailand OR Myanmar OR Philippines OR Cambodia OR Singapore OR Malaysia OR “southeast* asia” OR “south east asia” )) AND ( LIMIT‐TO ( DOCTYPE,“ar” ) ) AND ( LIMIT‐TO ( LANGUAGE,“English” ) )—822 articles.
